# Anti-inflammatory effects of clarithromycin in ventilator-induced lung injury

**DOI:** 10.1186/1465-9921-14-52

**Published:** 2013-05-10

**Authors:** Laura Amado-Rodríguez, Adrián González-López, Inés López-Alonso, Alina Aguirre, Aurora Astudillo, Estefanía Batalla-Solís, Jorge Blazquez-Prieto, Emilio García-Prieto, Guillermo M Albaiceta

**Affiliations:** 1 Servicio de Medicina Intensiva, Hospital Universitario Central de Asturias, Oviedo, Spain; 2 Departamento de Biología Funcional, Instituto Universitario de Oncología del Principado de Asturias (IUOPA), Universidad de Oviedo, Oviedo, Spain; 3 Departamento de Cirugía y Especialidades Médico-quirúrgicas, IUOPA, Universidad de Oviedo, Oviedo, Spain; 4 CIBER-Enfermedades Respiratorias, Instituto de Salud Carlos III, Madrid, Spain

**Keywords:** Mechanical ventilation, E-selectin, Macrolides, Neutrophil migration

## Abstract

**Background:**

Mechanical ventilation can promote lung injury by triggering a pro-inflammatory response. Macrolides may exert some immunomodulatory effects and have shown significant benefits over other antibiotics in ventilated patients. We hypothesized that macrolides could decrease ventilator-induced lung injury.

**Methods:**

Adult mice were treated with vehicle, clarithromycin or levofloxacin, and randomized to receive mechanical ventilation with low (12 cmH_2_O, PEEP 2 cmH_2_O) or high (20 cmH_2_O, ZEEP) inspiratory pressures for 150 minutes. Histological lung injury, neutrophil infiltration, inflammatory mediators (NFκB activation, *Cxcl2*, IL-10) and levels of adhesion molecules (E-selectin, ICAM) and proteases (MMP-9 and MMP-2) were analyzed.

**Results:**

There were no differences among groups after low-pressure ventilation. Clarithromycin significantly decreased lung injury score and neutrophil count, compared to vehicle or levofloxacin, after high-pressure ventilation. *Cxcl2* expression and MMP-2 and MMP-9 levels increased and IL-10 decreased after injurious ventilation, with no significant differences among treatment groups. Both clarithromycin and levofloxacin dampened the increase in NFκB activation observed in non-treated animals submitted to injurious ventilation. E-selectin levels increased after high pressure ventilation in vehicle- and levofloxacin-treated mice, but not in those receiving clarithromycin.

**Conclusions:**

Clarithromycin ameliorates ventilator-induced lung injury and decreases neutrophil recruitment into the alveolar spaces. This could explain the advantages of macrolides in patients with acute lung injury and mechanical ventilation.

## Background

Severe cases of acute lung injury are related to high morbidity and mortality rates. These cases often require invasive ventilatory support, which increases the risk of complications [1]. Among other effects, mechanical ventilation superimposes a mechanical stress within the lung tissue [2], and may induce an additional injury (ventilator-associated lung injury, VALI). In patients with the acute respiratory distress syndrome, protective ventilatory strategies have demonstrated a reduction in mortality [3], highlighting the relevance of this pathogenetic mechanism. However, there are no universally applicable ventilatory settings that minimize the risk of VALI in all patients [4].

There are no pharmacological strategies that have been successfully used to treat or prevent VALI. Recently, a survival benefit in patients receiving mechanical ventilation and macrolide therapy has been reported [5]. Similarly, several studies have shown better survival rates in patients with pneumonia treated with macrolides [6], compared to those receiving other antibiotics. These results have been confirmed even in cases of macrolide-resistant bacteria [7] and in ventilator-associated pneumonia [8].

The mechanisms responsible for these benefits of macrolides are unknown. Macrolides exert some anti-inflammatory effects including modulation of leukocyte recruitment [9], a shift in cytokine release towards anti-inflammatory molecules [10] or inhibition of the extracellular matrix remodeling by targeting matrix-metalloproteases [11].

Inflammation is one of the key steps required for ventilator-induced lung injury (VILI, the experimental counterpart of VALI) [12] and different anti-inflammatory drugs have shown a decrease in VILI [13,14]. Based on the anti-inflammatory properties of macrolides, we hypothesized that clarithromycin could exert some beneficial effects in a model of VILI by attenuating the inflammatory response. To test this hypothesis, we submitted mice treated with vehicle, clarithromycin or levofloxacin to different ventilatory strategies and studied the severity of lung injury and the extent of the inflammatory response. Finally, as macrolide treatment decreased leukocyte infiltration, the steps needed for cell recruitment were assessed.

## Materials and methods

### Animals

8–12 week-old C57/BL6 mice were used in all the experiments. Mice were kept under specific pathogens-free conditions, with full access to food and water, in 12-hour light/dark cycles. The experimental protocol was approved by the University Animal Research Ethics Committee.

### Experimental model

Mice were randomly assigned to receive one of three treatments previous to ventilation: Vehicle (Ringer’s lactate), clarithromycin (50 mg/kg per dose) or levofloxacin (50 mg/kg per dose). Two doses with a 12-hour interval between them were administered intraperitoneally. It has been reported that doses in the 25–100 mg/kg range result in clinically achievable drug levels in humans [15,16]. One hour after the second dose, mice were anesthetized with intraperitoneal ketamine and xylazin, a tracheostomy was performed, and they were connected to a mechanical ventilator. The animals were randomized to receive low (peak inspiratory pressure 12 cmH_2_O, PEEP 2 cmH_2_O, respiratory rate 100/min) or high (peak inspiratory pressure 20 cmH_2_O, PEEP 0 cmH_2_O, respiratory rate 50/min) pressures, using an Evita 2 Dura ventilator (Dräger, Lübeck, Germany). After 150 minutes of ventilation, a laparotomy was performed, the aorta sectioned and the animal sacrificed by exsanguination. Then the thorax was opened and the lungs removed. The left lung was fixated with intratracheal 4% formaldehyde, and immersed in the same fixative. The right lung was frozen at −80°C for subsequent analysis. In additional animals (n = 5 per group), a bronchoalveolar lavage was performed and the protein content of the bronchoalveolar lavage fluid (BALF) measured (BCA protein assay, Pierce, USA).

### Histological studies

After fixation, the left lung was included in paraffin, and a standard hematoxilin-eosin staining was performed in three lung sections, separated by 1 mm intervals. A previously described score was used to quantify the severity of lung damage [17]. To measure the neutrophil infiltration of lung tissue, additional sections were immunostained using an anti-myeloperoxidase antibody (Thermo, USA). The number of myeloperoxidase-positive cells in three randomly chosen high-power fields per section was counted and averaged. Activation of Nuclear factor κB (NFκB) was measured by counting the percentage of positive nuclei in histological sections immunostained with an anti-p65 antibody (Cell signaling, USA).

### Biochemical measurements

The right lung was homogenized in a lysis buffer (199 mM Tris pH 7.4, 150 mM NaCl, 1 mM EDTA, 1% sodium deoxycholate, 1% Triton X-100, 0.25% SDS, 1 mM sodium orthovanadate) with a protease inhibitors cocktail (Complete Mini, Roche, Switzerland). The samples were centrifuged and the supernatants collected and stored. Protein content was measured using a BCA assay (Pierce, USA).

Nuclear extracts from lung tissue were prepared as previously described [18]. Briefly, frozen tissues were homogenized in a cold buffer (10 mM Tris–HCl pH 8, 1.5 mM MgCl_2_, 10 mM KCl, 1 mM DTT and protease inhibitor cocktail), centrifuged and the pellets resuspended in the same buffer with 0.1% Triton X-100. After incubation, samples were centrifuged and the nuclear pellets resuspended in a buffer containing 20 mM Tris pH8, 25% glycerol, 0.4 M NaCL, 1.5 mM MgCl2, 0.2 mM EDTA, 0.5 mM DTT and protease inhibitor cocktail. After incubation, the nuclear extracts were finally centrifuged and the supernatants collected and frozen at −80°C.

Gene expression was studied by quantitative PCR as described [19]. Total RNA was extracted from tissue using Trizol (Sigma, Poole, UK) and isopropanol precipitation. Using this RNA, cDNA was synthesized (Enhanced avian HS RT-PCR kit, Sigma, Poole, UK) and quantitative real time PCR carried out in triplicate for each sample. Expression of *Cxcl2* and beta-actin (*Actb,* as a loading control) was measured using Taqman probes (Mm00436450_m1 and 4352341E respectively, Applied Biosystems, USA). Relative expression was computed according to manufacturer’s instructions.

For western blotting assays, samples were loaded in a 10% SDS-polyacrilamide gel and electrophoresed. The proteins were then transferred to a nitrocellulose membrane and incubated with primary antibodies against p65 (Abcam, UK), E-selectin (Abcam, UK), Intercellular adhesion molecule-1 (ICAM-1, Abcam, UK) or beta-actin (Santa Cruz Biotechnologies #SC1616, USA). The binding of primary antibodies was detected by using a peroxidase-linked secondary antibody and a chemoluminiscent reaction in a LAS-3000 camera (Fujifilm Life Science, USA). Actin was used as loading control.

Matrix metalloproteinases (MMP) -2 and −9 were measured by gelatin zymography, as previously described [20]. IL-10 was quantified using an ELISA (eBioscience, San Diego, USA), following manufacturer’s instructions.

### Statistical analysis

All the results are expressed as mean ± SEM. Differences among groups were evaluated using an ANOVA, including treatment and ventilatory strategy as factors. Paired post-hoc tests were done using Bonferroni’s correction when appropriate. A p value lower than 0.05 was considered significant.

## Results

60 animals were included in the study (8 mice/treatment group received low-pressure ventilation and 12 mice/treatment group received high-pressure ventilation). In 30 additional animals (5 per group), a BALF was performed at the end of the experiment. All the animals survived the experimental protocol.

### Clarithromycin ameliorates ventilator-induced lung injury

**Figure 1:**
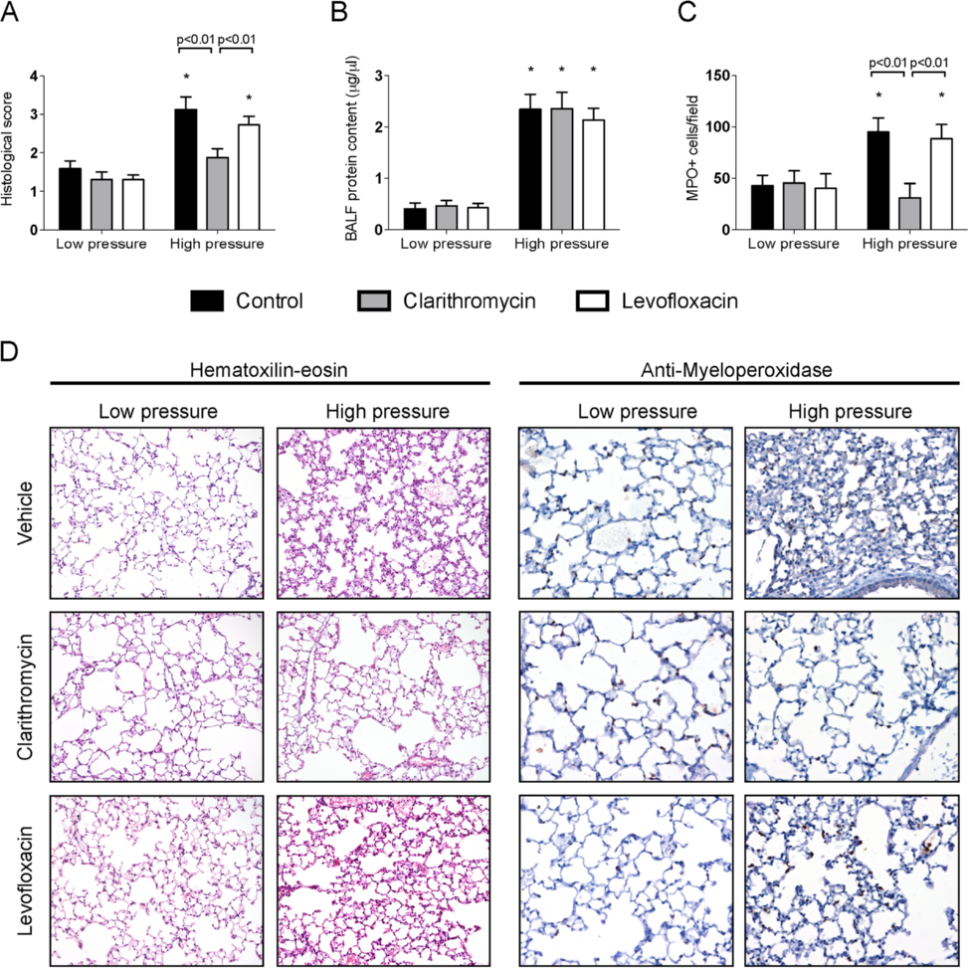
**Lung injury after mechanical ventilation.** Structural lung injury increased with high-pressure ventilation in vehicle- and levofloxacin-treated animals, but not in mice receiving clarithromycin (**A**). Alveolocapillary permeability, assessed by measurement of protein content in bronchoalveolar lavage fluid (BALF), increased after high-pressure ventilation irrespective of the treatment (**B**). Neutrophil recruitment increased after high-pressure ventilation in vehicle- and levofloxacin-treated animals. However, clarithromycin-treated animals showed no differences in neutrophil counts compared to mice ventilated with low pressures (**C**). Representative sections are shown in panel **D**. *p < 0.05 in post-hoc tests compared to low-pressure ventilated counterparts.

First, tissue injury was evaluated in histological sections (Figure 1A). Mechanical ventilation using low pressures and PEEP caused no histological injury within the lungs in any of the treatment groups. As expected, ventilation using high pressures and ZEEP was related to a significant increase in the lung injury score due to septal thickening and inflammatory infiltration. A similar increase was observed in vehicle- and levofloxacin-treated animals. However, clarithromycin-treated mice developed only a mild lung injury after ventilation.

Alveolocapillary permeability was assessed by measurement of protein content in BALF (Figure 1B). High-pressure ventilation increased the protein abundance, with no differences among genotypes.

Neutrophilic infiltration is one of the hallmarks of ventilator-induced lung injury. To confirm the decrease in inflammatory infiltrates observed in the hematoxylin-eosin-stained sections, an immunohistochemical study was performed. The amount of myeloperoxidase-positive cells (Figure 1C) was similar among the three groups of low-pressure ventilation. Neutrophil counts increased after high-pressure ventilation in vehicle and levofloxacin-treated animals. However, the number of neutrophils in clarithromycin-treated mice after VILI was similar to the cell counts observed after low-pressure ventilation. Figure 1D shows representative sections of each experimental group.

### Mechanisms of decreased neutrophilic infiltration

**Figure 2:**
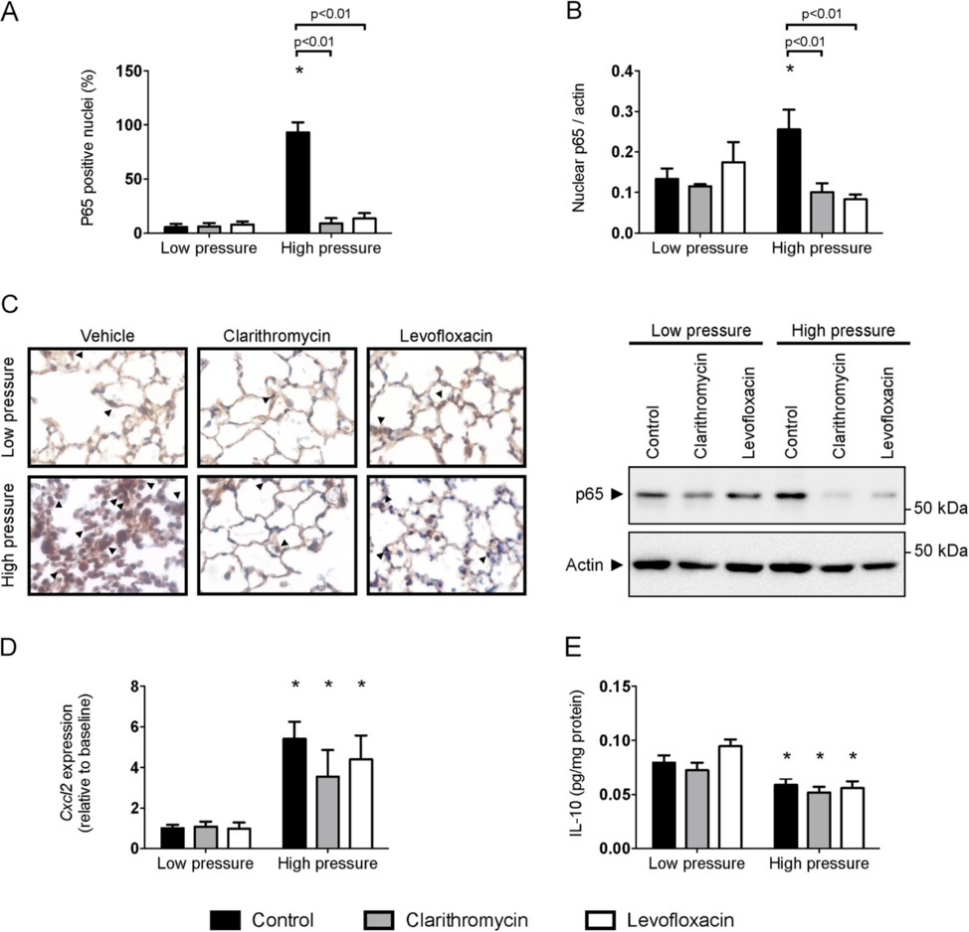
**Inflammatory response with high-pressure ventilation.** High pressure ventilation results in an increase in the activation of NFκB, measured as the percentage of p65 positive nuclei (**A**) or as the levels of p65 protein content in nuclear extracts (**B**). Both clarithromycin and levofloxacin blocked this activation. Panel **C** shows representative p65-immunostained sections and western blots of nuclear extracts. Some positive nuclei are shown (arrowheads). *Cxcl2* expression (**D**) and interleukin-10 levels were measured (**E**). There was a significant increase in *Cxcl2* expression and a slight decrease in IL-10 levels with injurious ventilation in all treatment groups. *p < 0.05 in post-hoc tests compared to low-pressure ventilated counterparts.

As the decreased neutrophilic count was the main difference among the three treatment groups, we focused on the steps of leukocyte recruitment. First, we studied activation of the inflammatory response by measuring the nuclear translocation of p65, a critical component of the NFκB pathway (Figure 2A-C). Injurious ventilation results in a highly significant increase in the percentage of p65 positive nuclei. However, levels of NFκB activation in clarithromycin- and levofloxacin-treated mice ventilated using high pressures were similar to their counterparts ventilated with low pressures. To confirm this finding, we measured levels of p65 in nuclear extracts. In line with the immunohistochemical findings, there was a lower p65 abundance in nuclei from clarithromycin- and levofloxacin-treated animals after injurious ventilation (Figure 2B). Panel 2C shows positive nuclei in all the experimental groups and a representative western blot of nuclear extracts.

Chemokines are inflammatory mediators involved in neutrophil recruitment. Expression of *Cxcl2* gene (corresponding to the chemokine MIP-2, the murine ortholog of IL-8) was measured in all the experimental groups. Mechanical ventilation increased its expression, but there were no significant differences among genotypes (Figure 2D). Additionally, we measured the levels of IL-10, an anti-inflammatory cytokine that could decrease neutrophil recruitment (Figure 2E). Ventilator-induced lung injury decreased IL-10 levels in lung homogenates compared to low-pressure ventilation groups, but there were no differences caused by treatment.

**Figure 3:**
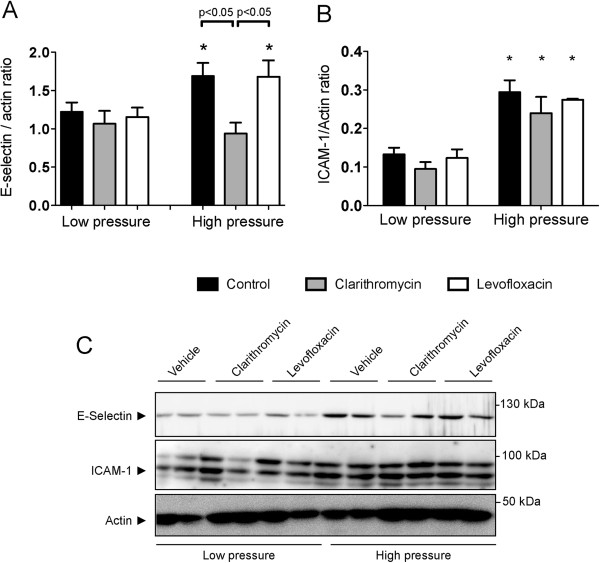
**Changes in adhesion molecules.** Mechanical ventilation using high pressures increased the levels of E-selectin (**A**) and ICAM-1 (**B**) in lung tissue homogenates. Clarithromycin dampened the increase in E-selectin after VILI. Representative western blots are shown in panel **C**. *p < 0.05 in post-hoc tests compared to low-pressure ventilated counterparts.

The next step for cell recruitment is the attachment of neutrophils to the endothelium to promote migration, so we measured E-selectin and ICAM-1 expression in lung homogenates (Figure 3). E-selectin levels were similar among groups after low-pressure ventilation, and increased after VILI in vehicle- and levofloxacin-treated mice. However, clarithromycin treatment dampened this increase in expression and protein levels in this group were similar to those found in animals ventilated with low pressure (Figure 3A). ICAM-1 increased in all the groups after injurious ventilation with no differences among treatment groups (Figure 3B). Panel 3C shows representative western blots.

**Figure 4:**
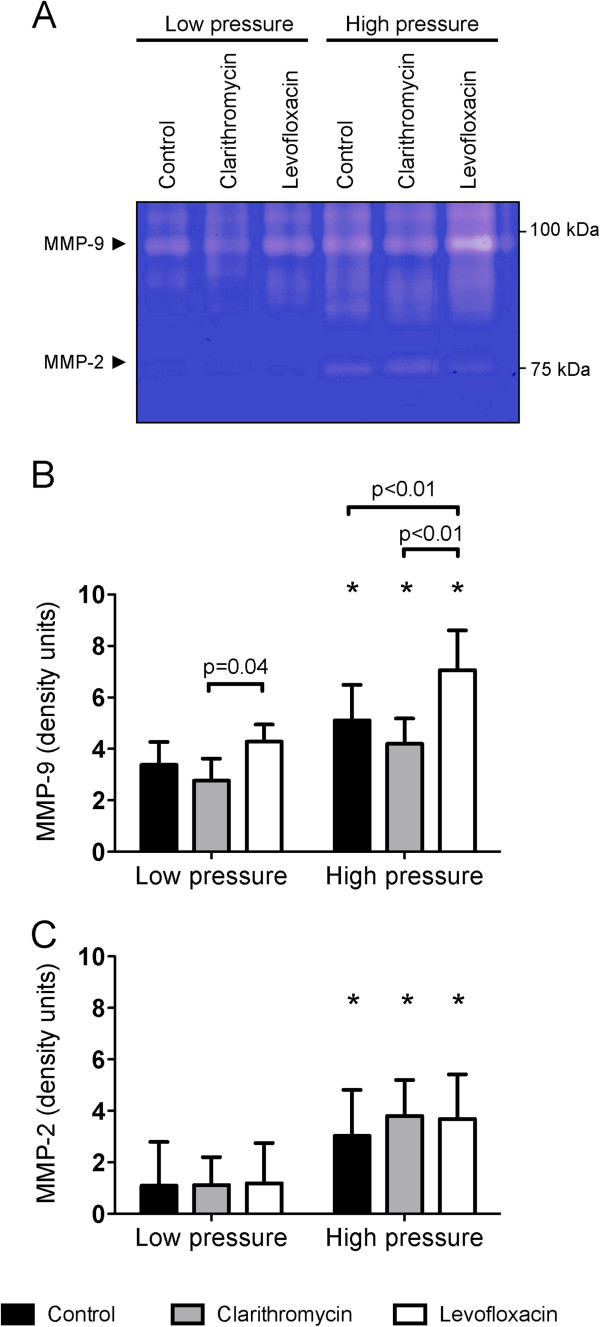
**Matrix metalloproteinase activity.** Both MMP-9 (**A**) and MMP-2 (**B**) increased in all the experimental groups submitted to high-pressure ventilation. MMP-9 levels were higher in levofloxacin-treated animals, compared to the other treatment groups and irrespective of the ventilatory strategy. A representative zymography is shown in panel (**C**).

Finally, we measured matrix metalloproteinases (Figure 4A), as its activity is required for the digestion of the extracellular fibers during cell migration. Matrix-metalloproteinase-9 is a gelatinase released mainly by neutrophils after activation. High-pressure ventilation increased MMP-9 levels in lung homogenates. Interestingly, levofloxacin-treated animals showed higher levels of MMP-9, irrespective of the ventilatory settings (Figure 4B). Matrix-metalloproteinase 2, which is a ubiquitous gelatinase, increased after VILI, but without differences among treatment groups (Figure 4C).

## Discussion

Our results show that pretreatment with clarithromycin ameliorates ventilator induced lung injury and decreases the lung neutrophilic infiltrate. This effect is related to a decrease in NFκB activity and E-selectin levels, that could be the mechanisms responsible for the decreased leukocyte recruitment.

Mechanical ventilation can induce or aggravate lung injury by a multifactorial mechanism, in which deformation of lung tissue triggers a complex biological response. Inflammation, extracellular matrix remodeling and different types of cell death have been described in response to mechanical ventilation [21]. Among these, alveolar inflammation with neutrophilic infiltration has been widely studied [22]. Different strategies aimed to decrease the polymorphonuclear infiltration within the lung have been demonstrated effective to decrease ventilator-induced lung injury [23]. Our results show a decreased neutrophilic infiltration in clarithromycin-treated animals, which could be the mechanism responsible for the beneficial effect of this macrolide. However, we did not find an improvement in alveolocapillary permeability. It has been proposed that the mechanisms that promote edema and cell infiltration could be independently regulated [24,25].

Macrolides are a family of antibiotics with immunomodulatory properties. Several studies have identified multiple mechanisms by which macrolides exert their anti-inflammatory effects. Using different models of acute lung injury (including LPS inhalation and bleomycin instillation), a decrease in neutrophilic lung infiltration has been demonstrated in clarithromycin-treated animals [26,27]. This effect could be mediated by several mechanisms. Activation of the NFκB pathway is one of the critical steps during a proinflammatory response. Our immunohistochemical findings show this activation to occur in isolated cells that could correspond to infiltrating leukocytes, although the lack of double immunostaining precludes any firm conclusion. It has been reported that both macrolides [28] and quinolones [29] may block NFκB activation, resembling our own results. However, we did not find any differences in *Cxcl2* expression that could explain the decreased neutrophilic infiltrates in animals receiving clarithromycin. Different intracellular mechanisms other than NFκB (such as MAP kinases) could be responsible for this increased chemokine expression [30].

Additionally, macrolides may decrease the levels of adhesion molecules such as ICAM-1, VCAM, E-selectin and P-selectin [31]. The increase in adhesion molecules during VILI has been described previously [32], and is needed for the attachment of neutrophils to the endothelium as an initial step for cell migration. Our results fit with these, showing an increase in ICAM-1 and E-selectin during VILI. However, mice treated with clarithromycin showed significantly lower levels of E-selectin after VILI. Lastly, although macrolides may also decrease MMP expression [11], we have found no differences in MMP-2 or −9 in our experimental model.

A wide range of strategies aimed to the limitation of the inflammatory response has shown positive results in models of VILI [13,14]. Although none of them have been translated to the clinical practice yet, a pharmacological strategy to ameliorate VALI could be a promising approach in ventilated patients.

Our results may have clinical implications that must be discussed. First, they could explain the beneficial effects observed in ventilated, macrolide-treated patients in both experimental models and the clinical practice. Moreover, clarithromycin (and probably other macrolides) could be used in ventilated patients only for its immunomodulatory effects. The recent finding of a decreased mortality in patients with acute lung injury receiving macrolide therapy [5] opens this possibility, as the benefit was independent of other variables such as severity scores, tidal volume or organ failures. The positive results observed in our study correlate with these studies showing an improved outcome in cases of pneumonia and/or septic shock receiving these drugs [8,33], and even with the use of macrolides to limit the inflammatory response in chronic lung diseases [34].

Our methodology has some limitations that must be clarified before any firm recommendation on macrolide use, especially with indications other than their antimicrobial properties. First, although widely used and accepted [35], our experimental model has no clear clinical correlate. The pressures and volumes used to induce VILI are higher than those used in the clinical practice. This was done to increase the signal-to-noise ratio, and precludes a direct translation of the findings. Second, no anti-inflammatory strategy has been shown effective to decrease mortality in patients with acute lung injury and mechanical ventilation. There are increasing evidences showing that inflammation is needed for later tissue repair [36-39]. In this sense, preventive or early inhibition of the inflammatory response may be beneficial, but a later inhibition could compromise lung healing. Therefore, these strategies should be viewed with caution and carefully studied. Third, use of antibiotics may have a profound impact on microbial populations and their sensitivities, and the benefits and risks of their application must include an epidemiologic approach before a systematic indication. Finally, we cannot discard other macrolide-triggered mechanisms, which could be responsible for the beneficial results shown here or even other unwarranted effects.

## Conclusions

In spite of these limitations, we may conclude that clarithromycin decreases VILI possibly by dampening the lung leucocyte infiltration. A decrease in NFκB activation and E-selectin expression could be the molecular mechanisms responsible for this effect. Although these results are subjected to the common limitations of preclinical studies, they give additional support to the use of macrolides in mechanically ventilated patients and open the possibility of a new therapeutic approach to limit ventilator-associated lung injury.

## Competing interests

The authors declare that they have no competing interests.

## Authors’ contributions

LA and GMA designed the protocol. LA, AGL, ILA, AA, JB, EGP and EBS performed the experiments. AAst made the histological studies. All the authors analyzed and discussed the data. LA and GMA wrote the manuscript. All authors read and approved the final manuscript.
